# Genomic and molecular landscape of homologous recombination deficiency across multiple cancer types

**DOI:** 10.1038/s41598-023-35092-w

**Published:** 2023-06-01

**Authors:** Zhiwen Shi, Bolin Chen, Xiao Han, Weiyue Gu, Shuzhi Liang, Lin Wu

**Affiliations:** 1 Department of Translational Medicine Center, Chigene (Beijing) Translational Medical Research Center Co., Beijing, 100176 China; 2 Obstetrics and Gynecology Hospital, NHC Key Laboratory of Reproduction Regulation, Shanghai Institute of Planned Parenthood Research, State Key Laboratory of Genetic Engineering at School of Life Sciences, Institute of Reproduction and Development, Fudan University, Shanghai, 200032 China; 3 Department of Thoracic Medical Oncology，Hunan Cancer Hospital/the Affiliated Cancer Hospital of Xiangya School of Medicine, Central South University, Changsha, 410000 China

**Keywords:** Cancer genomics, Cancer, Cancer microenvironment

## Abstract

Homologous recombination deficiency (HRD) causes faulty double-strand break repair and is a prevalent cause of tumorigenesis. However, the incidence of HRD and its clinical significance in pan-cancer patients remain unknown. Using computational analysis of Single-nucleotide polymorphism array data from 10,619 cancer patients, we demonstrate that HRD frequently occurs across multiple cancer types. Analysis of the pan-cancer cohort revealed that HRD is not only a biomarker for ovarian cancer and triple-negative breast cancer, but also has clinical prognostic value in numerous cancer types, including adrenocortical cancer and thymoma. We discovered that homologous recombination–related genes have a high mutation or deletion frequency. Pathway analysis shows HRD is positively correlated with the DNA damage response and the immune-related signaling pathways. Single cell RNA sequencing of tumor-infiltrating lymphocytes reveals a significantly higher proportion of exhausted T cells in HRD patients, indicating pre-existing immunity. Finally, HRD could be utilized to predict pan-cancer patients’ responses to Programmed cell death protein 1 immunotherapy. In summary, our work establishes a comprehensive map of HRD in pan-cancer. The findings have significant implications for expanding the scope of Poly ADP-ribose polymerase inhibitor therapy and, possibly, immunotherapy.

## Introduction

DNA damage is repaired through a network of interconnected pathways, one of which is the homologous recombination repair (HRR) pathway, the most precise and accurate DNA damage repair system responsible for double strand break (DSB) repair^1,2^. Homologous recombination deficiency (HRD) refers to the cellular level dysfunction of HRR. In the presence of HRD, DSBs become dependent on non-homologous end joining (NHEJ), microhomology mediated end joining (MMEJ)^3,4^, or low-fidelity and high-error-prone alternative DNA damage repair pathways such as single-strand annealing (SSA)^5^, which are likely to cause nucleic acid sequence insertion/deletion, abnormal copy number, and chromosomal cross-linking, resulting in genomic and chromosomal instability. HRD can be caused by many factors, including germline or somatic mutations in HRR-related genes, as well as epigenetic inactivation of HRR-related genes^6^. HRR is a multi-step signal transduction pathway in which the key protein is the breast cancer susceptibility gene (*BRCA*). It has been reported that carriers of germline *BRCA1/2* gene variants have an increased risk of breast, ovarian, pancreatic, and prostate cancer^7–9^. At present, new genes or mechanisms are still found to be involved in HRR regulation, such as *UBQLN4* and *RBBP8*^10,11^.

Tumor genome-specific alterations identified by HRD clinical testing are also referred to as “genomic scars.” Loss of heterozygosity (LOH)^12^, telomeric allelic imbalance (TAI)^13^, and large-scale state transition (LST) have been used as biomarkers to quantify the extent of genomic scars since 2012^14^. Three indicators, LOH, TAI, and LST, each of which has its own definition, may provide insight into the degree of cellular HRD status. Compared to a single index description, the comprehensive calculation score of the three can more precisely reflect the state of genomic scars and then evaluate the state of genomic instability^13,15^. The presence of HRD renders tumor cells more sensitive to platinum-based drugs that induce DNA cross-linking^16^ and augments the antitumor response to synthetic lethality of PARP inhibition (PARPi)^17^. HRD is currently being developed as an important biomarker for precision tumor treatment, and clinical detection of HRD is gaining popularity. Therefore, it's critical to investigate the clinical prognostic value of HRD as well as the changes in biological mechanisms caused by HRD in pan-cancer.

To gain a comprehensive understanding of HRD as a biomarker in Pan-cancer, we analyzed the genomic, epigenomic, and transcriptomic landscapes of HRD patients across 33 cancer types in The Cancer Genome Atlas (TCGA) database. We discovered that HRD has clinical prognostic value in a variety of cancer types, implying that the HRD could be used to identify patients who are likely to respond to platinum chemotherapy or PARPi. Using scRNA-seq and immunotherapy cohort data, we also identified that HRD is associated with tumor immunity and predicts immunotherapy response. The comprehensive analysis of HRD and its consequences in human cancer is provided below (Fig. 7). Both mechanistic and therapeutic investigations into the role of HRD in pan-cancer can be guided by our findings.

## Results

### Heterogeneity and clinical significance of HRD across patients with a given cancer type

**Figure 1:**
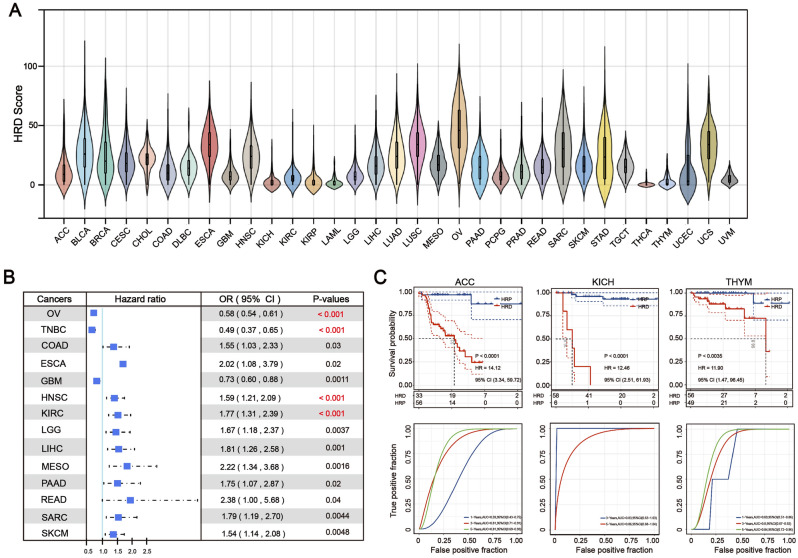
Illustrates the distribution of HRD scores across 33 different cancer types. (**A**) The median HRD scores for each cancer type are plotted. (**B**) The association between HRD and survival in the Pan-cancer cohort was delineated using a forest plot representation of the univariate Cox regression model. odds ratio: OR (**C**) Kaplan–Meier survival estimates for patients with HRD or HRP tumors in the ACC, KICH, and THYM cohorts. ROC curves and the corresponding AUC values for HRD in the ACC, KICH and THYM cohorts.

The median HRD score varied by more than a 100-fold between the 33 cancer types (Fig. 1A). The median HRD score for THCA and LAML is as low as 0 (roughly no change across the entire genome), whereas the median HRD score for OV, UCS, LUSC, and ESCA is over 30. Surprisingly, HRD scores varied significantly between patients with the same type of cancer. In OV, the frequency ranged between 1 and 99, whereas in UCS, it ranged between 2 and 77. in spite of the low median value (0) for LAML, patient-specific frequencies ranged from 0 to 20.

The distribution of HRD scores in various types of cancer follows a normal distribution, indicating that it can reflect the heterogeneity of tumors in different patients and therefore has the potential to serve as a molecular marker. Consistent with previous findings^18^, we discovered that patients with HRD had a favorable prognosis in OV (HR = 0.58, *P* < 0.001) and TNBC (HR = 0.49, *P* < 0.001) (Fig. 1B). Simultaneously, GBM patients with HRD have a significantly better prognosis than homologous recombination proficient (HRP) patients (HR = 0.73, *P* = 0.0012). In contrast, we discovered that HRD patients had a worse overall prognosis than HRP patients in other cancer types, including ACC (HR = 14.12, *P* < 0.001), KICH (HR = 12.46, *P* < 0.001) and THYM (HR = 11.90, *P* < 0.001) (Fig. 1C and Supplementary Table 6). Furthermore, as a prognostic factor, its predictive accuracy has improved over time: The high area under the precision-recall (AUC) curves (5 years, AUC = 0.81, 0.88, and 0.84, respectively) demonstrated HRD's excellent performance (Fig. 1C).

### The landscape of somatic genetic alterations in HRR-related genes across cancer types

**Figure 2:**
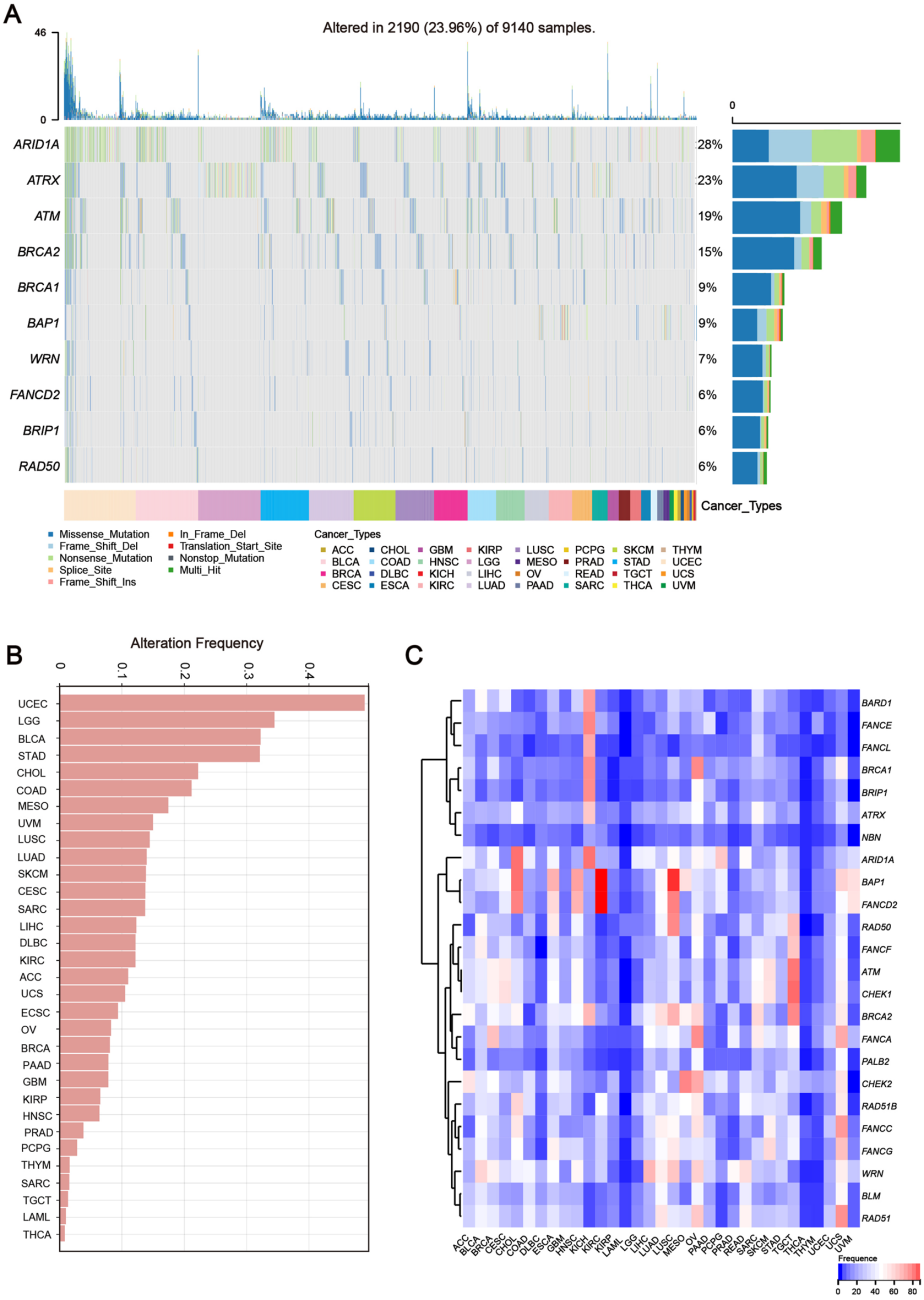
The landscape of somatic genetic alterations in HRR-related genes across cancer types. (**A**) Mutation landscape of HRR-related genes across different cancer types. Oncoprint plot on the left illustrates the overall frequency of mutations in HRR-related genes (rows, with gene names listed to the left) across 9140 samples. The color key at the bottom indicates the type of cancer. (**B**) Histogram showing the mutation frequency of HRR-related genes in different cancer types. (**C**) A heatmap illustrating the variable frequency of CNV deletion in different cancer types. Cancer types (columns) and genes associated with the HRR (rows).

Currently, it is known that genetic mutations and epigenetic inactivation of HRR-related genes can cause HRD. We began by calculating the mutation frequency and CNV (heterozygous deletion) in a pan-cancer cohort containing 33 distinct types of cancer. As previously described^19^, DNA alterations were classified as the following: missense, frame-shift, splice site, nonstop, nonsense, fusions, deletions, and changes in the translation start site. The mutation rate of HRR-related genes varied between 2 and 28% (Fig. 2A). Over half of the patients had at least one type of HRR-related gene mutation (Fig. 2A). *ARID1A* was the most frequently mutated HRR-related gene, followed by *ATRX*, *ATM*, and *BRCA1/2*. The mutation frequency of HRR-related genes was increased in UCEC, BLCA and LUSC (Fig. 2B and Supplementary Fig. 1A). The mutation landscape of HRR-related genes revealed several possible recurrent hotspot driver mutations in *ARID1A*, *ATRX*, *ATM*, and *BRCA1/2*, including R1989* in *ARID1A*, which was carried by over 30 tumor patients (Supplementary Fig. 1B).

To identify the CNV alteration, the SNP array data of HRR-related genes from the TCGA database were analyzed. The CNV heatmap distribution revealed that the deletion of HRR-related genes is a frequent occurrence in Pan-cancer. CNV analysis indicated that heterozygous deletion of *ARID1A* was prevalent in ESCA and KICH; *BRCA2* CNV deletion was more common in Pan-cancer than *BRCA1* CNV deletion. Furthermore, the frequency of HRR-related gene deletion in UCS, OV, LUSC, and KICH was significantly higher than in other cancers (Fig. 2C). The high frequency of somatic alterations in HRR-related genes suggests that the HRR signaling pathway and tumorigenesis are linked. The landscape of methylation in HRR-related genes also revealed an abnormal methylation signature of HRR-related genes such as *ARID1A*, whose methylation levels were significantly higher in tumor tissues compared to normal tissues (Supplementary Fig. 2A).

### Gene expression analysis of HRD patients reveals up-regulation of DDR and immune-related signatures across cancers

**Figure 3:**
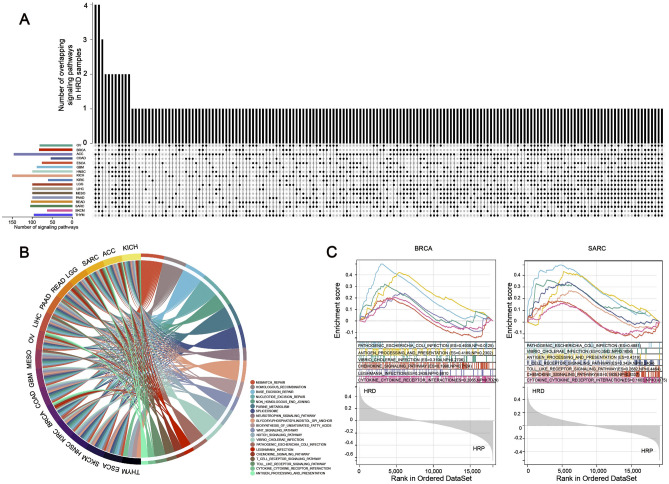
HRD correlated pathways and immunomodulators in the given cancer type. (**A**) The UpSetR plot depicts the overlap of GSEA pathways enriched for up-regulated genes in various cancers. (**B**) Chord plot illustrating the relationship between cancer types and signaling pathways that overlap. Different colors were used to indicate signaling pathways that crossed. (**C**) GSEA revealed that in BRCA and SARC, immune-related signaling pathways were upregulated in the HRD group compared to the HRP group. ES: enrichment score; NP: Nominal *P* value.

To advance our understanding of the biology of HRD tumors, GSEA was performed on each cancer type to investigate HRD-associated pathways, with a particular emphasis on up-regulated signaling pathways. The UpSetR plot demonstrated the overlapped of transcriptomic changes in HRD tumors with various types of cancer (Fig. 3A). As a result, we discovered that DNA damage response (DDR) pathways such as mismatch repair and homologous recombination pathways were positively associated with HRD in more than 16 cancer types, confirming that DDR maintained genome integrity by detecting damage and activating a complex signaling network that promotes DNA repair (Fig. 3B). Intriguingly, we observed that HRD tumors activate a large number of immune-related pathways. HRD tumors activate pathways such as toll-like receptor signaling, chemokine signaling, and infection-related immune signaling in many cancers of epithelial origin (BRCA, ESCA, SARC, OV, KICH, and ACC) (Fig. 3B). As illustrated in Fig. 3C, these immune-related pathways were up-regulated in the HRD group compared to the HRP group in BRCA and SARC. It has been reported that in cancer cells with HRD, the DNA substrates generated by HRD cannot be resolved, triggering the release of genomic DNA from the nucleus to the cytoplasm and activating cytosolic DNA-sensing and innate immune responses. According to the UpSetR map, the hub genes of these up-regulated immune-related signal pathways in HRD patients overlap with cytosolic DNA-sensing system genes (Supplementary Fig. 2B). Furthermore, the correlation heatmap revealed that type I IFN expression, which is one of the downstream targets of the cytosolic DNA-sensing pathway, was linked to a higher HRD score in a variety of cancer types, including BRCA, GBM, OV, and THYM (Supplementary Fig. 2C).

### Underlying extrinsic immune landscapes of HRD patients

**Figure 4:**
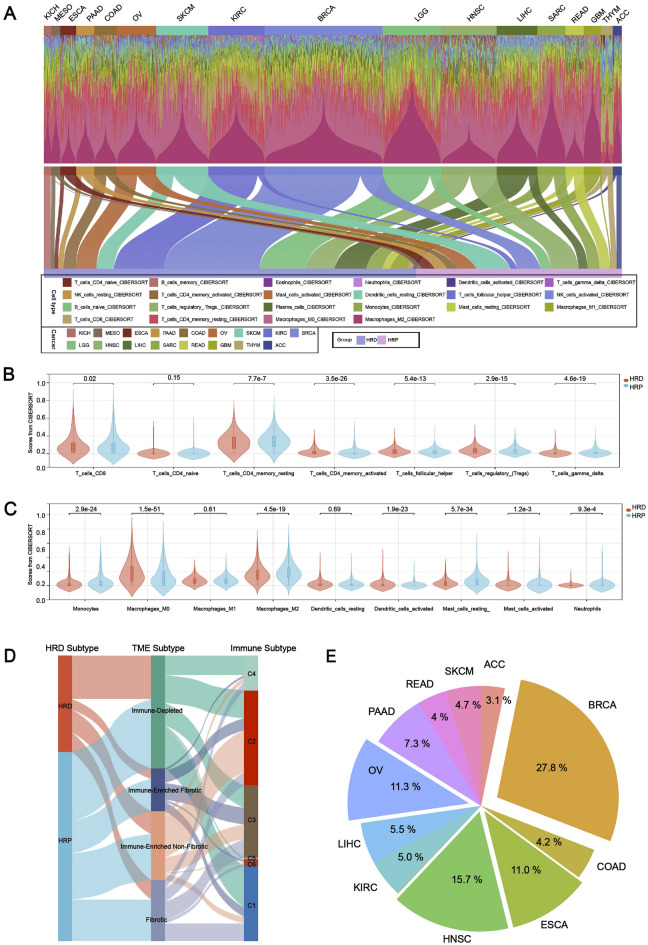
Immune profiles of the HRD patients in the TCGA cohort. (**A**) Bar charts illustrating the proportions of 22 different types of immune cells estimated using the CIBERSORT method from RNA sequencing data for each patient, and a Sankey diagram illustrating how the TCGA cohort was classified into HRD and HRP groups. (**B**) Comparison of T lymphocytes estimated using the CIBERSORT method using RNA sequencing data from the HRD and HRP groups (Wilcoxon signed-rank test). (**C**) Comparison of myeloid cells estimated using the CIBERSORT method using RNA sequencing data from the HRD and HRP groups (Wilcoxon signed-rank test). (**D**) Sankey plot comparing HRD and HRP patients (left) per TME subtype to immune subtypes (right) across all TCGA patients. C1: wound healing; C2: IFN-g dominant; C3: inflammatory; C4: lymphocyte depleted; C5: immunologically quiet; C6: TGF-b dominant. (**E**) Pie charts were used to depict the proportions of various cancer types within the IM subtype.

Transcriptome analysis revealed that immune-related signaling pathways were activated in HRD patients, implying that their tumor microenvironment (TME) may differ from that of HRP patients. To gain a better understanding of the relationship between the TME and HRD in Pan-cancer, we analyzed the immune infiltration of patients with CIBERSORT (Fig. 4A). Comparing the two groups showed that the HRD group had a higher proportion of T lymphocytes than the HRP group (Fig. 4B). Specifically, the proportion of immune-stimulatory cells (including follicular helper T cells, CD4^+^ memory activated and CD8^+^ T cells) was significantly higher in the low-risk group than in the high-risk group (Wilcoxon signed-rank test, *P* < 0.001 and *P* < 0.01, respectively) (Fig. 4B). The signals of tumor-infiltrating myeloid cells also vary significantly between the HRD and HRP groups. The proportion of M2 and Mast cells in the HRP group was significantly higher than in the HRD group, while the proportion of activated dendritic cells was significantly lower (*P* < 0.001) (Fig. 4C).

To further validate the above findings, we analyzed the TME differences between the HRD and HRP groups using the Alexander et al. TME algorithm and the Thorsson et al. Immune-subtype algorithm^20,21^. TME analysis results demonstrated that nearly half of HRD patients were classified as Immune-Depleted, which was consistent with the findings from the melanoma immunotherapy cohort^22^. The majority of the remaining HRD patients were classified as Immune-Enriched Fibrotic/non-Fibrotic (IM) (Fig. 4D). Around 80% of HRD patients were classified as C2 (IFN-dominant) using the Immune-subtype algorithm. The results of the Immune subtype analysis matched those of the correlation heat map analysis, indicating that tumors with high HRD scores had high type I IFN expression (Supplementary Fig. 2C). BRCA, HNSC, OV, ESCA, and PAAD are the top five cancer types in terms of patient numbers in the IM subgroup (Fig. 4E). In addition, the distribution of immune landscape in HRD and HRP groups for a number of cancer types with larger cohorts was analyzed. Intriguingly, the distribution of immune subtypes varies greatly between different types of cancer. For instance, in breast cancer, the proportion of immune-Enriched subtype in the HRD group is greater than 50%, whereas in HNSC, it is less than 15%. (Supplementary Fig. 3A).

### Single-cell RNA sequencing elucidates the biology of HRD tumors in BRCA and the tumor-infiltrating T cells in KIRC

**Figure 5:**
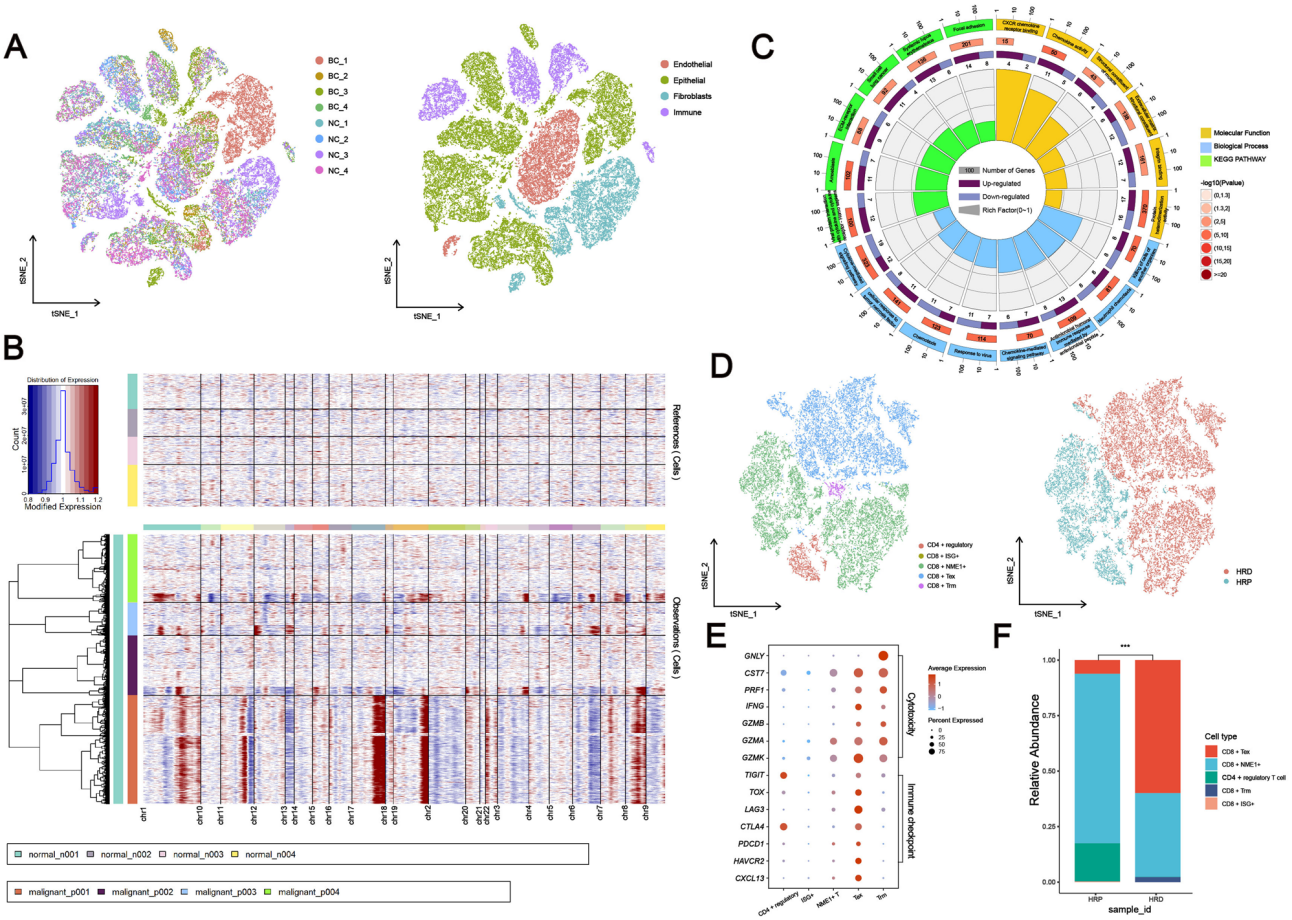
Single-cell RNA sequencing of HRD tumors. (**A**) tSNE is based on the top twenty principal components of all single-cell transcriptomes after filtering, and is color-coded according to tissue type or primary cell type. (**B**) The chromosomal landscape of inferred large-scale copy number variations (CNVs) differentiates malignant from benign cells. Amplifications (red) and deletions (blue) were inferred by averaging expression across 100-gene segments on the respective chromosomes. (**C**) KEGG and Gene Ontology enrichment analysis of DEGs. The outermost ring represented the signaling pathways’ names; the second outer ring represented the number of genes in signaling pathways; the column heights in the inner ring indicated the proportion of DEGs in the total number of genes in the signaling pathway; and the color depth indicated the number of differential genes. (**D**) tSNE calculated from the top ten principal components of tumor-infiltrating lymphocytes after filtering, and color-coded according to tissue type or major cell type. (**E**) Dot plot demonstrating the expression of five tumor-infiltrating lymphocytes’ signature genes. (**F**) Quantification of tumor-infiltrating lymphocytes in HRD or HRP patients (Fisher extract test, *P* < 0.01).

To study the cellular biology of HRD tumors, we analyzed single-cell sequencing data from four normal breast tissues and four breast cancer tissues with *BRCA1* pathogenic mutations that were collected during surgery. After quality control and filtering, 55,463 high-quality transcriptomes were obtained (Sample information was listed in Supplementary Table 3). Analysis and visualization by t-Distributed Stochastic Neighbor Embedding (tSNE) showed that single-cell transcriptomes of different tissue types or patients intermingled in many clusters and partly formed tumor- or patient-specific clusters, indicating underlying biological differences (Fig. 5A). We classified single cells into breast epithelial cells (*KRT8/18*, *ACTA2*, *CNN1*), immune cells (*PTPRC* +), fibroblasts (*DCN*), and endothelial cells (*PECAM1*) based on previous research^23^ (Fig. 5A and Supplementary Fig. 3B). Epithelial transcriptomes were then subsetted and reclustered to better understand interpatient variability within the breast epithelial cell compartment. Comparing proportion of cells in a cluster to all epithelial cells for tumor and normal separately: clusters overrepresented in normal samples are supposed to be cells of normal breast epithelial cells, all other clusters are supposed to be malignant cells (Supplementary Fig. 3C), which was largely congruent with the copy-number status of cells (Fig. 5B). Malignant cell clusters were segregated from normal cell clusters and were mainly patient-specific, indicating intertumoral heterogeneity. Gene Ontology analysis demonstrated that DEGs between malignant and normal cells are enriched in immune-related signals (Fig. 5C), similar to the results of GSEA analysis of bulk RNA-seq data from TCGA samples: that is, HRD tumors upregulate the immune-related signaling pathway.

To further characterize T lymphocytes in HRD tumors further, we analyzed scRNA-seq data from tumor-infiltrating T lymphocyte suspensions extracted from HRD and HRP specimens in KIRC. T lymphocytes were classed as CD8^+^ (ISG^+^, NME1^+^, Tex, Trm) and CD4 ^+^ regulatory by scType algorithm (Fig. 5D). CD8 + Tex cells were characterized by expression of both cytotoxicity marker genes, such as *GZMA/B/K* and *IFNG*, and immune checkpoint marker genes, such as *LAG3* and *PDCD1* (Fig. 5E). The proportions of tumor-infiltrating lymphocytes were then compared between HRD and HRP tumors. As shown in Fig. 5F, when HRD tumors were compared to HRP tumors, CD8 ^+^ Tex cells were significantly increased (60% vs. 6.2%, *P* < 0.001) and CD4 ^+^ regulatory cells were significantly decreased (0.1% vs 17.2%, *P* < 0.001).

Recent evidence indicates that terminal Tex cells in tumors are derived specifically from tumor-specific T cells^24,25^, whereas T cells responsible for acute infections do not produce Tex cells^26^. Consequently, a terminal Tex subset can serve as a proxy for a compartment of tumor-reactive T cells^27^. Importantly, the data provide direct evidence that intratumoral T cells in the patients with HRD were distinct from those in the patients with HRP.

### Data from mouse model suggest that HRD might serve as a prognostic marker for immunotherapy

To determine if HRD can serve as a predictive biomarker of immunotherapy response, HRD was examined in a well-validated mouse model of mammary tumors^28–31^. As shown in Table 1, in the absence of immunotherapy, the median survival time of mice in the HRD group was nearly double that of the HRP group (Supplementary Table 7). With the administration of immunotherapy, this discrepancy became even more pronounced. And when the tumor is HRD, further augmentation of the tumor's genomic instability (such as overexpression of *Apobec3* or UV irradiation) can boost the immunotherapy's effectiveness.

**Table 1:** Response statistics to combination immunotherapy in mouse models.

PDX cell name	Genetics background	HRD subgroup	Median survival untreated	Median survival aPD1/aCTLA4	Response
2225L	TP53^−/−^	HRP	10	9.5	Resistant
2336R	TP53^−/−^	HRP	19	18	Resistant
2224L	TP53^−/−^	HRP	9	9	Resistant
9263-3F	TP53^−/−^	HRP	9	9	Resistant
KPB25L	Brca1 f.^/f^	HRD	21	28	Sensitive
KPB25L-Apobec	Brca1 f.^/f^; Apobec3				
Overexpressed	HRD	43	64	Sensitive	
KPB25L-UV	Brca1 f.^/f^; Short-wave UV exposure	HRD	37	74	Sensitive

**Figure 6:**
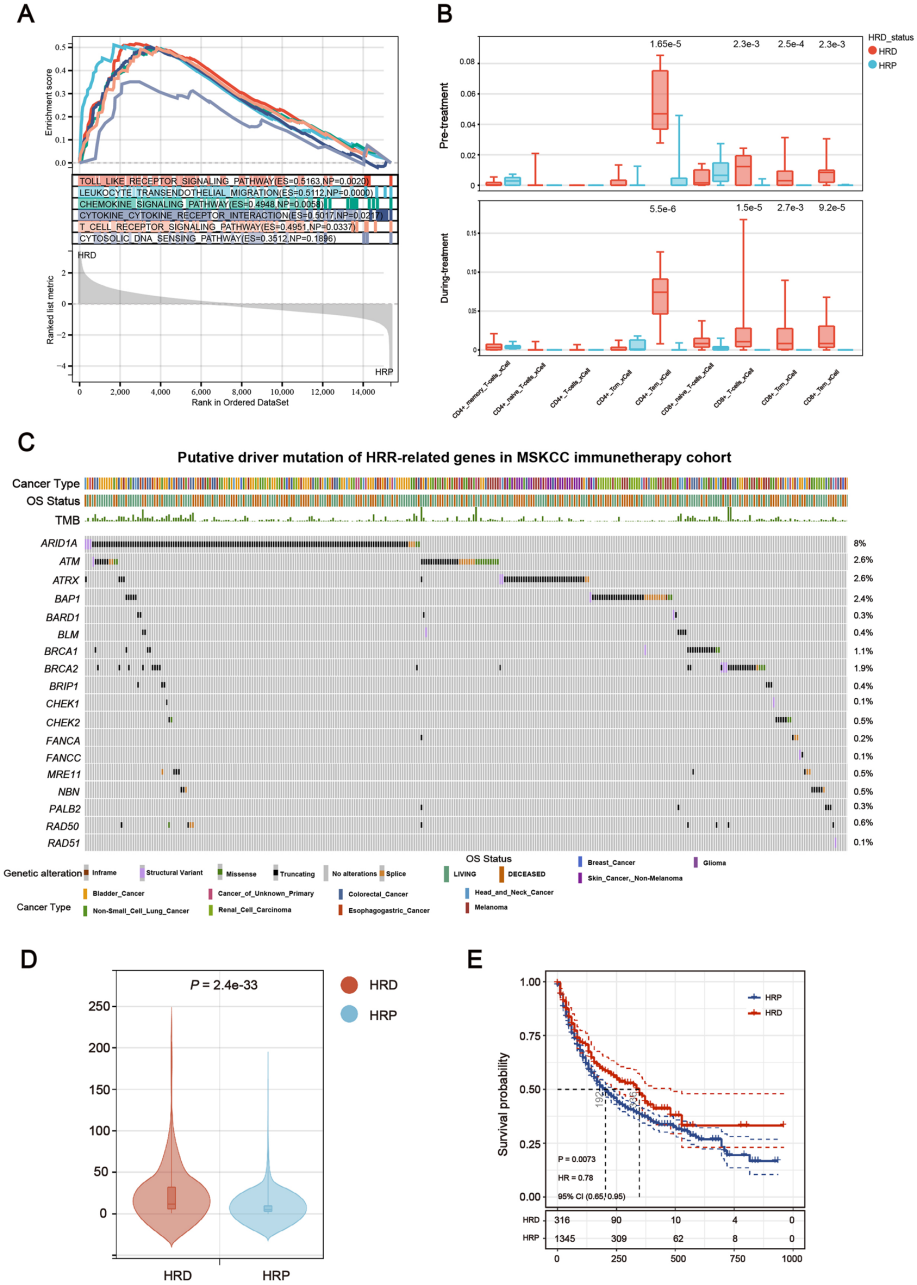
Immunotherapy could be beneficial in the treatment of HRD tumors. (**A**) GSEA showed that compared with HRP group, the immune related signal pathway in HRD group was up-regulated in mouse model. (**B**) Comparison of T lymphocytes estimated using the xCell method from the HRD and HRP groups (Wilcoxon signed-rank test). (**C**) HRR-related gene driver mutation landscape in an MSKCC immunotherapy cohort. Oncoprint plot displays the overall frequency of deleterious mutations, deletions, and epigenetic silencing events for each significantly silenced HRR-related gene. Cancer type is shown in the color key to the bottom. (**D**) Violin plot of TMB in the HRD or HRP group (Wilcoxon signed-rank test, *P* < 0.001). (**E**) Kaplan–Meier survival curves for OS of MSKCC immunotherapy cohort in the HRD group versus the HRP group. Log rank test *P* values are displayed in the bottom left-hand corner of the plot.

Using transcriptome data, we discovered that innate immune signals, such as the chemokine signaling pathway and the cytosolic DNA sensing pathway, are enriched in the HRD subtype (Fig. 6A), which is consistent with our analysis of bulk RNA and scRNA data from patient tumor tissue. Using the xCell technique, we next calculated the scores for the nine T lymphocyte subtypes in order to examine the link between HRD subgroup and immune cell invasion. Before and throughout immunotherapy, the HRD group had a considerably increased amount of lymphocytes, including CD8 + T cells, CD4 + Tem cells, CD8 + Tcm cells, and CD8 + Tem cells (Fig. 6B). These findings show that HRD might be a crucial indicator of immunotherapy success in the mouse models investigated here.

### Immunotherapy could be beneficial in the treatment of patients with HRD

Previously published clinical research has established a link between immunotherapy response, particularly immune checkpoint blockade, and T cell infiltration^32,33^, high tumor mutational burden (TMB)^34,35^, neoantigen burden^36^, and TME^37^. To test the clinical value of HRD as a biomarker for predicting response to immunotherapy, we examined the clinical outcomes of HRD patients treated with immune checkpoint inhibitors. We obtained complete clinical, tumor-normal paired sequencing data of 1,661 patients across 11 different cancer types from the MSKCC database^35^. These patients were either treated with PD-1/PD-L1 inhibitors or with CTLA-4 blockade, or with a combination of immunotherapy and chemotherapy (Supplementary Table 2). To determine whether a patient's tumor tissue was HRD or HRP, we examined the mutational status of HRR-related genes in these patients: those with HRR-related gene driver mutations were classified as HRD, whereas the remaining patients were classified as HRP. As demonstrated in Figs. 6C, ~ 20% of patients were classified as having HRD. This frequency is comparable to previous research on the mutation rate of HRR-related genes in pan-cancer^38^. The most frequently observed HRR-related variants in this cohort were *ARID1A* mutations (8%), followed by *ATM* (2.6%), *ATRX* (2.6%), and *BAP1* (2.4%). The driver mutation was predominantly an inactive truncating mutation, which makes sense given that all HRR-related genes are tumor suppressor genes (Figs. 6C). According to a comparison of TMB between the two groups, HRD patients had significantly higher TMB than HRP patients, which was confirmed in the TCGA cohort (Figs. 6D and Supplementary Fig. 3D). Furthermore, compared to HRP patients in the cohort, HRD patients had a significantly longer overall survival (OS) (Figs. 6E, P = 0.0073, hazard ratio (HR) = 0.78, 95% confidence interval (CI) = 0.65–0.95).

## Discussion

Several studies have explored HRD in various cancer types. However, prior research has the following limitations:

1. The lack of clinical prognostic information and insufficient cancer type coverage ^39^.
2. HRD is defined by the presence of known pathogenic variants in HRR-related genes, which rules out HRD caused by epigenetic alterations or other unknown causes^40^.
3. Previous pan-cancer studies on HRD patients focused solely on genomics, with no in-depth research on other omics, such as the transcriptome and TME^41^.
4. Up until now, HRD has been used primarily as a marker of genomic instability in order to facilitate the use of platinum and PARP inhibitors, and its relevance to immunotherapy has not been extensively studied^42^.

**Figure 7:**
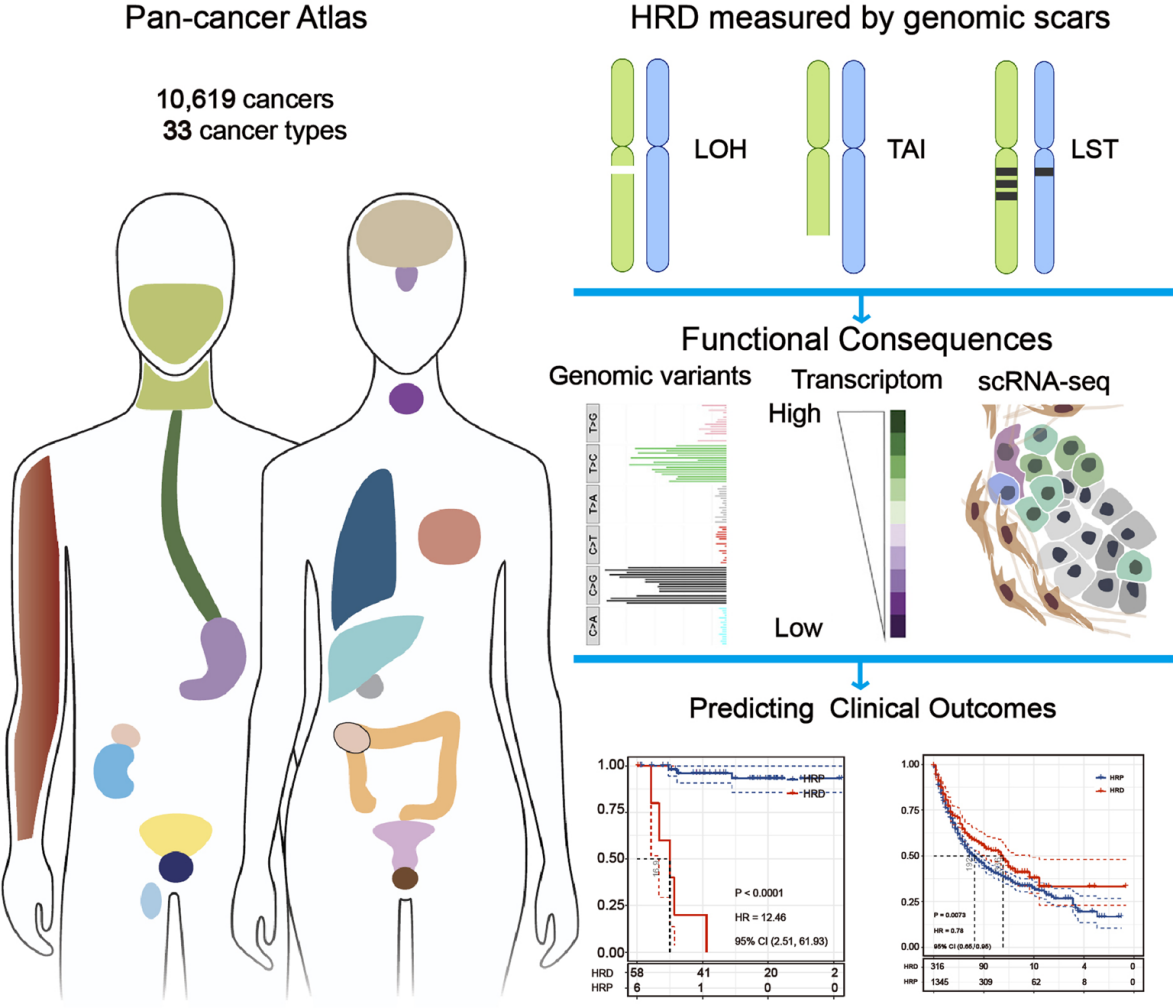
TCGA Pan-Cancer analysis of HRD in cancer. Through integrative genomic and molecular analyses, frequent HRD alterations are identified across 33 cancer types. This work also demonstrates the correlation between gene- and pathway-level alterations of HRD. Furthermore, the prognostic utility of HRD is highlighted, providing valuable insights into the potential prognostic value of HRD in cancer.

By analyzing 10,619 tumors representing 33 different cancer types, we examined the clinical significance and biological characteristics of HRD in Pan-cancer. These findings establish the largest clinical reference resource for HRD research (Fig. 7). We demonstrate that HRD is not only prevalent in ovarian and breast cancer, but also occurs frequently in other epithelial malignancies, such as LUSC, LUAD, and SARC. The prevalence of HRD across cancer types may indicate the existence of a distinct but identifiable subpopulation of cancer patients who could benefit from genotoxic therapy but are not currently receiving it as standard of care.

We discovered shared activated signaling pathways in HRD patients with various cancer types through GSEA. The widespread activation of DDR signaling pathways supports the notion that HRD serves as a biomarker for assessing genomic instability. Additionally, HRD patients exhibit activation of immune-related signaling pathways, including microbial infection and immune chemokine signaling pathways. To confirm that these activated immune-related signaling pathways originate from HRD tumor cells, we conducted scRNA-seq analyses. The results of scRNA-seq directly proved that HRD tumor cells up-regulated the immune-related signaling pathways in BRCA. Endogenous DNA has been shown to activate innate immune responses, which were originally characterized as the first line of defense against pathogens^43,44^. Genotoxic stress, induced by inactivation of HRR-related genes, results in the formation of chromosomal fragments that are recognized by the nucleic acid sensor cyclic GMP-AMP (cGAMP) synthase^45,46^. Furthermore, activation of innate immune signals causes changes in TME, as evidenced by our immune cell enrichment signals and scRNA-seq results. While the scRNA-seq data were derived from a small number of patients, they replicate the cohort-level findings and provide additional evidence for immunotherapy in HRD patients. However, it is important to note that the TME subtype of the tumors of many HRD patients is immune-depleted. Recent research has demonstrated that activation of STING signaling results in the expansion of Breg cells with immunosuppressive properties. Breg cells promote tumor growth by inducing the formation of immunosuppressive TME by secreting IL-10 and IL-35 in response to the activation of STING signaling^47^.

The observation that HRD is associated with increased TMB and immune-related signaling pathways, which lends credence to the possibility of an expansion of the immune-responsive patient population^42,44^. The scientific rationale for PARPi and immunotherapy is related to immune activation, not only because error-prone repair may result in an increase in point mutations and neoantigen load, but also because innate cytosolic DNA can activate type I immunity via the cGAS-STING pathway^48^. Additionally, several critical HRR pathway genes, such as *ATM*, *ATR*, and *CHK1*, play critical roles in cell cycle regulation, which can result in an increase in programmed death-ligand 1 expression (PD-L1)^49,50^. And in breast and ovarian cancer, PARPi increases PD-L1 expression in tumor cells. These indications suggest that some cancer patients may benefit from the combination of PARPi and immunotherapy^51–53^.

We propose a model based on published data and our findings that elucidates the mechanism by which HRD activates the cGAS-STING pathway, thereby facilitating immunotherapy. In a cohort of 1661 patients undergoing immunotherapy for 11 different cancer types, we examined the association between HRD and immunotherapy. The findings indicated that immunotherapy-treated HRD patients had a significantly better prognosis than immunotherapy-treated HRP patients. Given that some HRP patients have pathogenic HRR-related gene mutations defined as VUS or epigenetic variants in the HRR-related genes, the clinical significance of HRD as an immunotherapy prognostic marker may be underestimated. Further research should be conducted to assess HRD status on a genome-wide scale to determine whether HRD can be used effectively as a predictive biomarker for patients who may benefit from combination therapy with DNA damaging agents and immune checkpoint inhibitors.

To summarize, our findings establish a critical benchmark for the standardization of HRD detection, and its application prospects are promising. In the future, with the rapid advancement of genetic testing technology, the continuous improvement of HRD evaluation methods, and the involvement of an increasing number of clinicians, pathologists, molecular testing personnel, clinical pharmacists, and tumor biology experts in tumor precision medicine, we believe that accurate HRD assessment will further improve the level of tumor diagnosis and treatment, benefiting more tumor patients.

## Materials and methods

### Data collection and processing

We obtained Affymetrix SNP6 genotyping data and for 10,619 unique cancer samples representing 33 distinct cancer types from the TCGA data portal (https://portal.gdc.cancer.gov). The genotyping data for TCGA from Affymetrix SNP assay used the hg19. Patients’ clinical information, RNA sequencing data (as TPM units, the version of genecode for gene annotation is genecodeV22), somatic mutation data and corresponding copy number variation (CNV) data were captured from the USCS XENA portal https://xenabrowser.net/datapages/?cohort=TCGA%20PanCancer%20(PANCAN)&removeHub=https%3A%2F%2Fxena.treehouse.gi.ucsc.edu%3A443, which are listed in Supplementary Table 1. The DNA sequencing data and corresponding clinical follow-up information from immunotherapy cohorts were extracted from Memorial Sloan Kettering Cancer Center (MSKCC) https://www.cbioportal.org/study/summary?id=tmb_mskcc_2018^35^, which are listed in Supplementary Table 2. Patients with multiple tumor RNA-Seq samples or clinical annotation gaps were eliminated. The sample information of scRNA-seq data were listed in Supplementary Table 3.

### HRD score analysis

Pairs of tumor and normal samples were normalized and preprocessed with the Aroma Affymetrix CRMAv2 algorithm^54^. The B-allele fraction (BAF) was adjusted with the CalMaTe and Tumor Boost algorithms, and the number of B-alleles was changed with the Tumor Boost algorithm^55^. The HRD score that includes NtAI, LST, and LOH (Supplementary Table 1).

### Somatic mutation and copy number variation (CNV) analysis

The HRR-related genes were downloaded from Molecular Signatures Database^56^. The following are the specific genes: *ARID1A, ATM, ATRX, BAP1, BARD1, BLM, BRCA1, BRCA2, BRIP1, CHEK1, CHEK2, FANCA, FANCC, FANCD2, FANCE, FANCF, FANCG, FANCL, MRE11A, NBN, PALB2, RAD50, RAD51, RAD51B, WRN*^38^. CNV data was extracted from the TCGA database and analyzed using web tools for 33 cancer types (http://bioinfo.life.hust.edu.cn/GSCA/#/)^57^. Supplementary Table 9 shows the mutation landscape of HRR-related genes.

### Methylation analysis

Illumina Human Methylation 450 k-level 3 methylation data were obtained from UCSC Xene database (https://xenabrowser.net/datapages). The methylation signature of HRR-related genes was analyzed by GSCA web tools (http://bioinfo.life.hust.edu.cn/GSCA/#/).

### Survival analysis and receiver operating characteristic curves calculation

We conduct univariate survival analysis using the R package survival. Survival differences were assessed using log-rank test^58^. The straightforward method for determination of a prognostic cutoff point is to optimize the significance of the split in the Kaplan–Meier plot. R package PRROC that were used to estimate the ROC curve^59^.

### GSEA analysis

GSEA was used to identify differential signaling pathways in different groups using GSEA software from the Broad Institute (MIT, Cambridge, MA)^60^. The plots of the overlapping GSEA results were created using the R package UpSetR^61^.

### Evaluation of immune infiltration with CIBERSORT

CIBERSORT is a deconvolution algorithm that is based on gene expression and uses support vector regression to infer cell type proportions in data from mixed cell type cancer samples^62^. Based on normalized gene expression data (TPM), the proportions of different types of infiltrating immune cells were estimated using the CIBERSORT method (Permutations = 200) or × Cell^63^. The reference signature immune cell type for CIBERSORT is in (Supplementary Table 8).

### Evaluation of TME and Immune subtype

A tumor's four TME subtypes identified using the classification platform's TME subtypes. The four TME subtypes are: Immune-enriched, fibrotic (IE/F), Immune-enriched (IE), Fibrotic (F), Immune-Depleted (D)^64^. The six immune subtypes were retrieved from the immune landscape publication^65^. The TME and Immune subtypes of each sample are detailed in Supplement Table 4. The input matrix was quantified as TPM; Scripts used to generate results are available at https://github.com/BostonGene/Kassandra ; https://github.com/CRI-iAtlas .

### scRNA-seq data processing and quality control

We conducted pre-processing of scRNA-seq fastq files using Cell Ranger (10 × Genomics), aligning the reads to the GRCh38 reference genome and generating a count matrix of cell barcodes by genes using the Cell Ranger count function. To normalize the number of confidently mapped reads per cell across libraries from different samples, we used the “Cell Ranger Aggr” function. Poor-quality cells were excluded based on specific criteria, such as a low number of detected genes (< 500) or a high number of detected genes (> 10,000), a low number of unique molecular identifiers (UMI) (< 1000) or a high number of UMIs (> 100,000), and a high percentage of molecules mapped to mitochondrial genes (≥ 10%)^66^. To remove heterotypic doublets, we preprocessed the dataset using DoubletFinder v2.0.2^67^ (assuming 6% of barcodes represent doublets). After filtering, we normalized the library with SCTransform^68^, We conducted principal component analysis (PCA) on all single-cell transcriptomes using genes expressed in at least two cells. To correct for batch effects, we used Harmony^69^. We then applied the k-means algorithm to cluster cells based on the PCA results, and visualized cell distances in a reduced two-dimensional space using the t-distributed stochastic neighbor embedding (t-SNE) method. Cell type annotation was conducted by using scType^70^ and the cell markers used in this work were extracted from previous studies^71^ (Supplementary Table 5). To identify differentially expressed genes (DEGs) between two groups of clusters, we used edgeR^72^ to evaluate the significance of each gene (FDR < 0.01; fold change |log2FC|> 1).

## Data and code availability

The RNA-seq data of Patient-Derived Xenograft (PDX) model are available at GEO Datasets: GSE124821, GSE136206. The single cell datasets generated during this investigation are accessible through the Zenodo database (https://zenodo.org/record/7905511#.ZFhcunZBwQ8). Source of the original data are provided with this paper. The study did not produce any new bioinformatics methods, the code supporting the current study is available from the corresponding authors on request.

## Supplementary Information

Supplementary Information 1.

Supplementary Information 2.

Supplementary Information 3.

Supplementary Information 4.

Supplementary Information 5.

Supplementary Information 6.

Supplementary Information 7.

Supplementary Information 8.

Supplementary Information 9.

Supplementary Information 10.

Supplementary Information 11.

Supplementary Information 12.

## Abbreviations

SNP: Single-nucleotide polymorphism
HRD: Homologous recombination deficiency
scRNA-seq: Single-cell sequencing
HRR: Homologous recombination repair
DSB: Double strand break
NHEJ: Non-homologous end joining
MMEJ: Microhomology mediated end joining
SSA: Single-strand annealing
BRCA: Breast cancer susceptibility gene
LOH: Loss of heterozygosity
TAI: Telomeric allelic imbalance
LST: Large-scale state transition
PARP: Poly ADP-ribose polymerase
TCGA: The cancer genome atlas
MSKCC: Memorial Sloan Kettering cancer center
BAF: B-allele fraction
CNV: Copy number variation
PDX: Patient-derived xenograft
PD-1: Programmed cell death protein 1
TPM: Transcripts per kilobase million
IE/F: Immune-enriched, fibrotic
IE: Immune-enriched
F: Fibrotic
D: Immune-depleted
HRP: Homologous recombination proficient
AUC: Precision-recall
TME: Tumor microenvironment
IM: Immune-enriched fibrotic/non-fibrotic
tSNE: T-distributed stochastic neighbor embedding
TMB: Tumor mutational burden
OS: Overall survival
HR: Hazard ratio
CI: Confidence interval
SNVs: Single nucleotide variants
VUS: Variants of unknown clinical significance
cGAMP: Cyclic GMP-AMP
PD-L1: Programmed death-ligand 1 expression
LAML: Acute myeloid leukemia
ACC: Adrenocortical carcinoma
BLCA: Bladder urothelial carcinoma
BLCA: LGG brain lower grade glioma
BRCA: Breast invasive carcinoma
CESC: Cervical squamous cell carcinoma and endocervical adenocarcinoma
CHOL: Cholangiocarcinoma
LCML: Chronic myelogenous leukemia
COAD: Colon adenocarcinoma
ESCA: Esophageal carcinoma
GBM: Glioblastoma multiforme
HNSC: Head and neck squamous cell carcinoma
KICH: Kidney chromophobe
KIRC: Kidney renal clear cell carcinoma
KIRP: Kidney renal papillary cell carcinoma
LIHC: Liver hepatocellular carcinoma
LUAD: Lung adenocarcinoma
LUSC: Lung squamous cell carcinoma
DLBC: Lymphoid neoplasm diffuse large B-cell lymphoma
MESO: Mesothelioma
MISC: Miscellaneous
OV: Ovarian serous cystadenocarcinoma
PAAD: Pancreatic adenocarcinoma
PCPG: Pheochromocytoma and paraganglioma
PRAD: Prostate adenocarcinoma
READ: Rectum adenocarcinoma
SARC: Sarcoma
SKCM: Skin cutaneous melanoma
STAD: Stomach adenocarcinoma
TGCT: Testicular germ cell tumors
THYM: Thymoma
THCA: Thyroid carcinoma
UCS: Uterine carcinosarcoma
UCEC: Uterine corpus endometrial carcinoma
UVM: Uveal melanoma
