# Supplementary figures and images for: Genomic and molecular landscape of homologous recombination deficiency across multiple cancer types
Source: Sci Rep. 2023 Jun 1;13:8899. doi: 10.1038/s41598-023-35092-w (PMC10235055; doi:10.1038/s41598-023-35092-w)

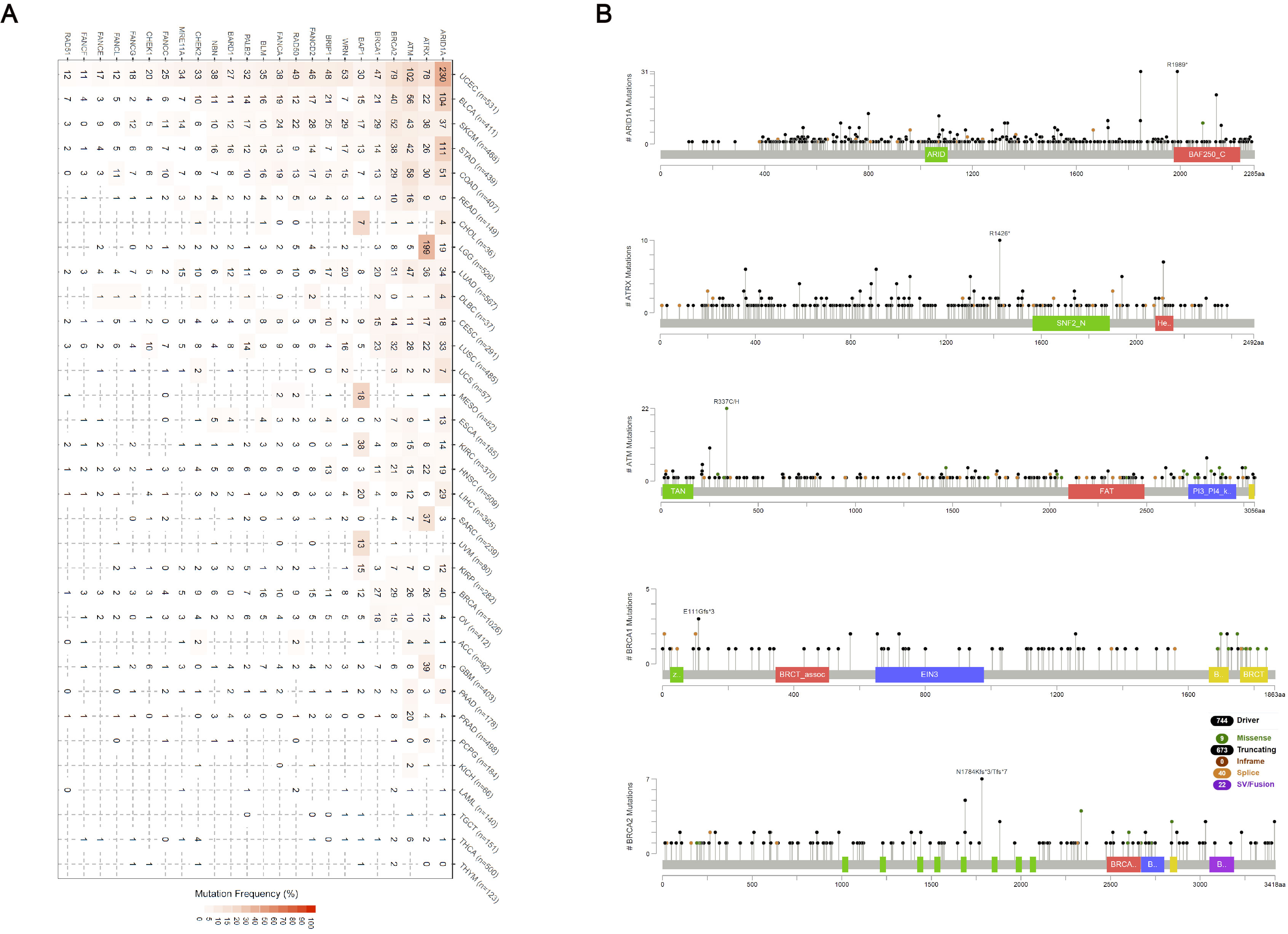

Supplement: Supplementary file 1 — Supplementary Information 1. [file 41598_2023_35092_MOESM1_ESM.jpg]

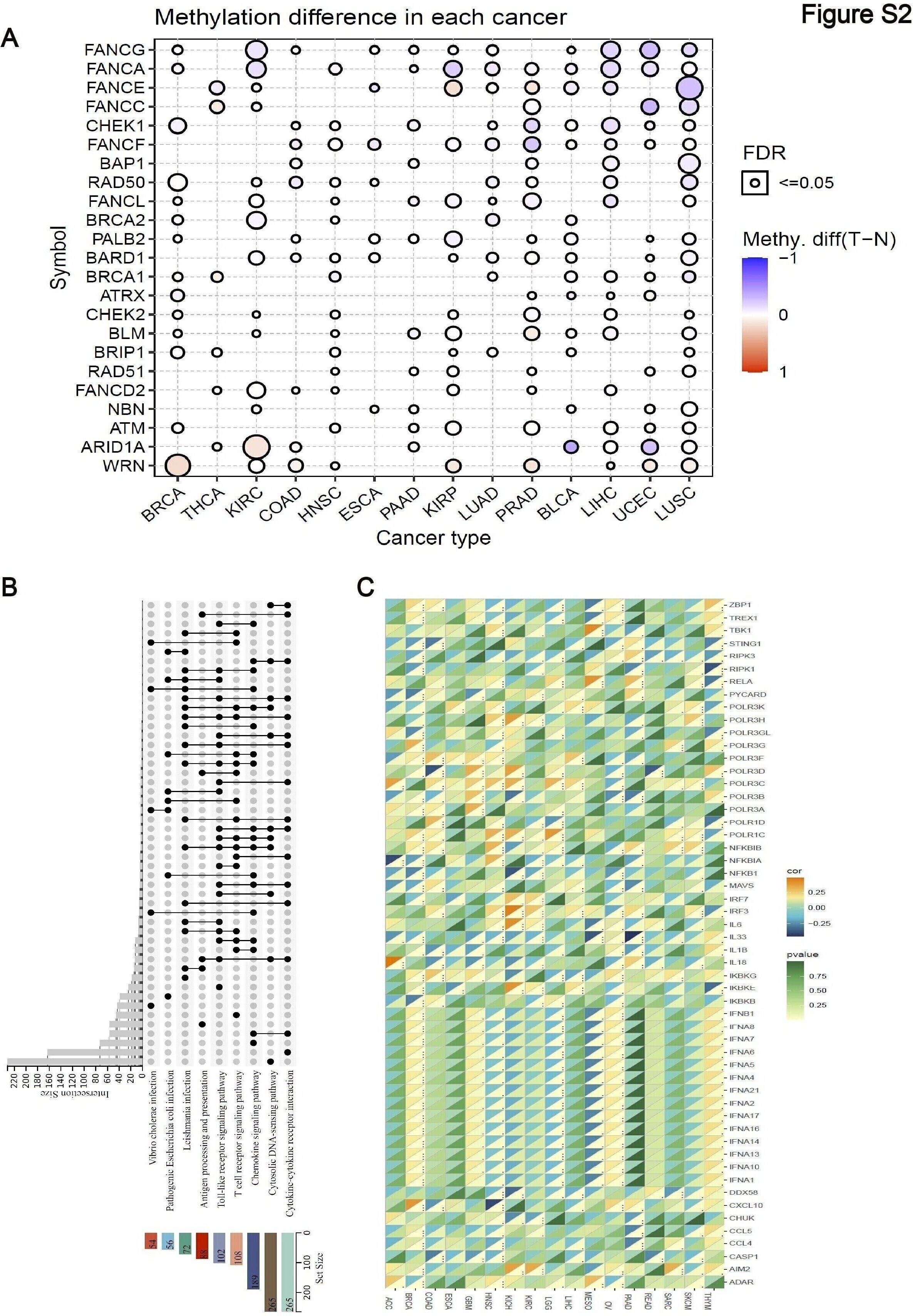

Supplement: Supplementary file 2 — Supplementary Information 2. [file 41598_2023_35092_MOESM2_ESM.jpg]

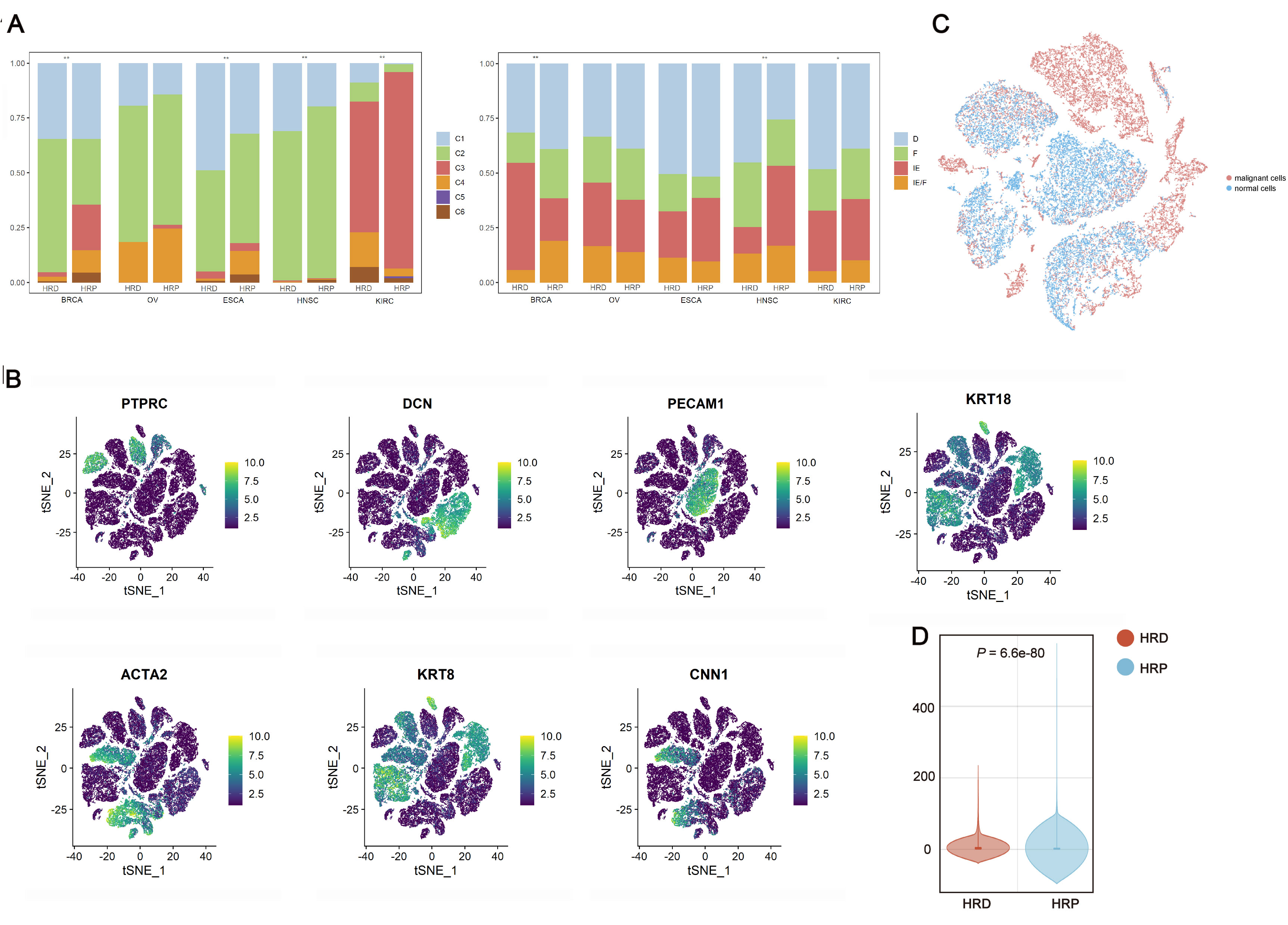

Supplement: Supplementary file 3 — Supplementary Information 3. [file 41598_2023_35092_MOESM3_ESM.jpg]
